# ABCG2 Protein Levels and Association to Response to First-Line Irinotecan-Based Therapy for Patients with Metastatic Colorectal Cancer

**DOI:** 10.3390/ijms21145027

**Published:** 2020-07-16

**Authors:** Jesper Andreas Palshof, Camilla Natasha Cederbye, Estrid Vilma Solyom Høgdall, Tim Svenstrup Poulsen, Dorte Linnemann, Sune Boris Nygaard, Jan Stenvang, Ib Jarle Christensen, Benny Vittrup Jensen, Per Pfeiffer, Nils Brünner, Mette Yilmaz, Birgitte Martine Viuff, Dorte Lisbet Nielsen

**Affiliations:** 1 Department of Oncology, Herlev and Gentofte Hospital, University of Copenhagen, Herlev Ringvej 75, DK-2730 Herlev, Denmark; jesper.andreas.palshof@regionh.dk (J.A.P.); benny.vittrup.jensen@regionh.dk (B.V.J.); dorte.nielsen.01@regionh.dk (D.L.N.); 2 Faculty of Health and Medical Sciences, Department of Drug Design and Pharmacology, Section for Molecular Disease Biology University of Copenhagen, 2200 N Copenhagen, Denmark; camilla@cederbye.dk (C.N.C.); stenvang@sund.ku.dk (J.S.); bmv842@gmail.com (B.M.V.); 3 Department of Pathology, Herlev and Gentofte Hospital, University of Copenhagen, Herlev Ringvej 75, DK-2730 Herlev, Denmark; estrid.hoegdall@regionh.dk (E.V.S.H.); tim.svenstrup.poulsen@regionh.dk (T.S.P.); dorte.linnemann@regionh.dk (D.L.); ib.jarle.christensen.02@regionh.dk (I.J.C.); 4 Department of Pathology, Rigshospitalet, University of Copenhagen, Blegdamsvej 9, DK-2100 Copenhagen Ø, Denmark; snyg@dadlnet.dk; 5 Scandion Oncology, Fruebjergvej 3, DK-2100 Copenhagen, Denmark; 6 Department of Oncology, Odense University Hospital, Sdr. Boulevard 29, DK-5000 Odense C, Denmark; per.pfeiffer@rsyd.dk; 7 Department of Oncology, Aalborg University Hospital, Hobrovej 18-22, DK-9100 Aalborg, Denmark; m.yilmaz@rn.dk

**Keywords:** biomarker, colorectal cancer, ABCG2 protein, BCRP, irinotecan

## Abstract

In this study we investigated the use of cancer cell protein expression of ABCG2 to predict efficacy of systemic first-line irinotecan containing therapy in patients with metastatic colorectal cancer (mCRC). From a Danish national cohort, we identified 119 mCRC patients treated with irinotecan containing therapy in first-line setting. Among these, 108 were eligible for analyses. Immunohistochemistry (IHC) analyses were performed on the primary tumor tissue in order to classify samples as high or low presence of ABCG2 protein. Data were then associated with patient outcome (objective response (OR), progression free survival (PFS) and overall survival (OS)). ABCG2 protein expression in the basolateral membrane was high (score 3+) in 33% of the patients. Exploratory analyses revealed a significant interaction between ABCG2 score, adjuvant treatment and OR (*p* = 0.041) in the 101 patients with evaluable disease. Patients with low ABCG2 (score 0–2) and no prior adjuvant therapy had a significantly higher odds ratio of 5.6 (Confidence Interval (CI) 1.68–18.7; *p* = 0.005) for obtaining OR. In contrast, no significant associations between ABCG2 expression and PFS or OS were found. These results suggest that measurement of the ABCG2 drug efflux pump might be used to select patients with mCRC for irinotecan treatment. However, additional studies are warranted before conclusions regarding a clinical use can be made. Moreover, patients with high ABCG2 immunoreactivity could be candidates for specific ABCG2 inhibition treatment in combination with irinotecan.

## 1. Introduction

Worldwide, colorectal cancer (CRC) ranks in the top of cancer-related morbidities and mortalities with an estimated 1.3 million new cases and almost 700,000 deaths from CRC every year [1]. Predictive CRC biomarkers are only available for epidermal growth factor receptor (EGFR) targeting drugs where testing for mutational status of the RAS genes is mandatory [2]. Predictive biomarker candidates have been tested for the efficacy of 5-flurouracil (5-FU), irinotecan and oxaliplatin, but none have reached a level of evidence allowing for clinical routine use [3].

Irinotecan or oxaliplatin in combination with 5-FU yields similar response rates of approximately 50% when used as first-line treatment for metastatic (m)CRC [4,5]. This implies that a substantial group of mCRC patients does present with chemotherapy resistant disease and will therefore not gain any treatment benefit but may only experience drug-induced adverse effects. Equally important is the lack of complete cross-resistance between these drug combinations [5]. During the course of an ineffective treatment, the patient may deteriorate thus making later lines of therapy impossible. This underlines the importance of selecting the most effective drug combination already in first line.

ABCG2 is a potential predictive biomarker for irinotecan. ABCG2 is a member of the ATP-binding cassette (ABC) transporter family. As a major drug transporter of substrates across extra- and intra-cellular membranes, ABCG2 overexpression has been associated with multidrug resistance in various cancer diseases [6,7]. Preclinical studies from our group have shown that acquired SN-38 (the active metabolite of irinotecan) resistance in CRC cell lines may associate with increased *ABCG2* mRNA and protein expression and that specific inhibition of ABCG2 can restore SN-38 sensitivity [8]. This is in accordance with results published by others [9,10,11]. Using mRNA expression data from patients enrolled in the PETACC-3 study [12], it appeared that in the adjuvant setting of stage III colon cancer increased *ABCG2* mRNA expression together with low Topoisomerase 1 (*TOP 1*) gene expression were associated with decreased benefit from the addition of irinotecan to 5-FU [13]. However, conflicting results are published for the association between efflux transporters, including ABCG2, and clinical outcome in various solids tumors [9,14,15,16,17].

Another important implication of ABCG2 mediated irinotecan resistance is the use of potential ABCG2 inhibitors combined with irinotecan [18]. A number of drugs with both ABCB1 and ABCG2 inhibitory activity have been presented [14,16,17] but due to either unacceptable toxicity from the inhibitor or to problematic clinical trial designs [14], no definite conclusions on the efficacy of these inhibitors can be drawn. No specific ABCG2 inhibitors have yet been tested in the clinical setting.

Based on our previous studies, and data published by others, we raised the hypothesis that mCRC patients having low basolateral membrane ABCG2 immunoreactivity in their tumor cells benefit the most from irinotecan containing therapy.

## 2. Results

### 2.1. Patients and Outcome

**Figure 1:**
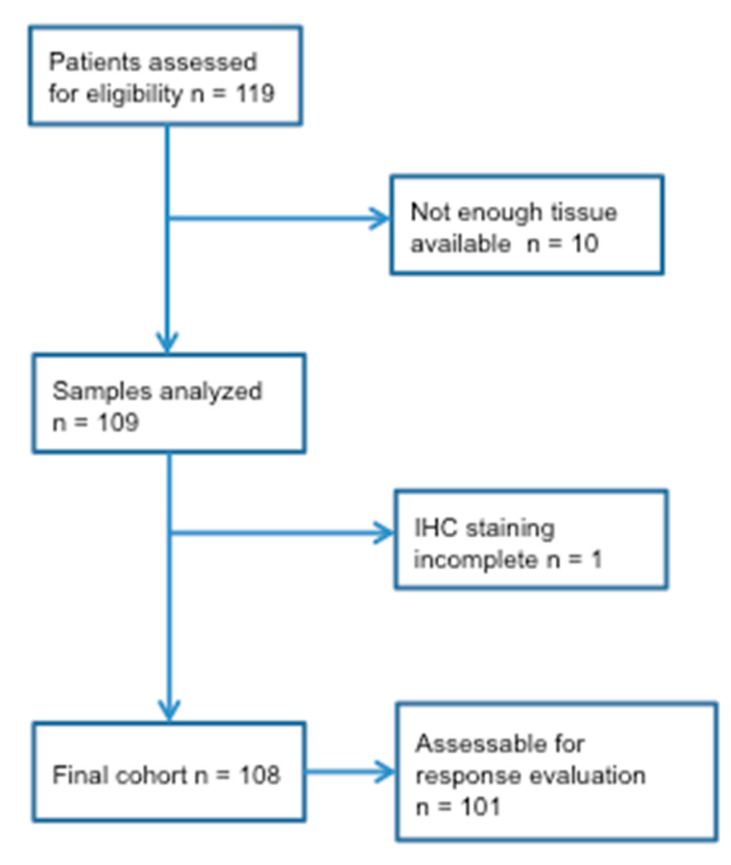
CONSORT diagram showing the flow of patients and samples.

Of the 119 consecutive patients who received irinotecan in first-line therapy, 10 patients were excluded as they did not have enough tissue available for analyses. In one patient (1%) immunohistochemistry (IHC) staining was incomplete. Thus, 108 patients were eligible for biomarker analyses (Figure 1). Patient characteristics are shown in Table 1.

The 108 patients were treated with the following regimens as first-line therapy: 82 (76%) received a combination of 5-fluorouracil/leucovorin (5-FU/LV) and irinotecan. Thirteen (12%) received irinotecan as monotherapy, eight patients (7%) capecitabine + irinotecan, and five patients (5%) received triplets therapy consisting of 5-FU/LV irinotecan and bevacizumab.

Median PFS was 7.8 months (range: 1.5–44.9) and median OS was 25.3 months (range: 4.2–132.7). Response evaluation was assessable for 101 patients. PR was seen in 42.5% (43/101) and non-response in 57.5% (58/101). Stable disease was recorded as the best result in 42.5% (43/101) and 15 patients (15%) were recorded with progressive disease.

### 2.2. ABCG2

**Figure 2:**
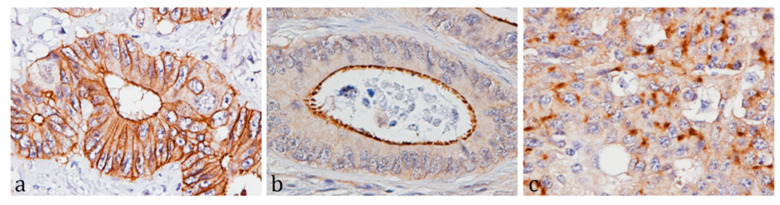
Immunostaining of ABCG2 in colorectal cancer (CRC) tissue demonstrating the three distinguishable ABCG2 immunoreactivity patterns in the cancer cells. ABCG2 was detected using the HiDef DetectionTM HRP Polymer system, visualized with 3,3’-diaminobenzidine-tetrahydrochloride (DAB+), and counterstained with Mayer’s hematoxylin, 40× magnification. (**a**) Basolateral membrane immunostaining, (**b**) apical membrane immunostaining, (**c**) cytoplasmic immunostaining.

Three distinguishable ABCG2 expression patterns of the tumor cells could be detected, namely basolateral membrane, apical membrane and cytoplasmic immunostaining (Figure 2). The expression patterns could be detected separately or in combination, therefore, a separate score was given for each pattern using the guidelines described in [19].

Overall, 72 (67%) were scored as ABCG2 low (score 0–2) and 36 (33%) as ABCG2 high (score 3) when using the basolateral membrane score. Fourteen tumors (13%) were scored as 0, 18 (16%) tumors as 1+, 40 (37%) tumors as 2+, and 36 (33%) tumors as 3+.

The association between ABCG2 basolateral membrane score and baseline characteristics did not reveal any significant associations, except when comparing whether the primary tumor had been resected or not (*p* = 0.03) (Table 1).

In the logistic regression analysis including the 108 evaluable patients, a non-significant trend between ABCG2 and OR was demonstrated. An odds ratio of 2.14 (CI 0.86–5.35; *p* = 0.10) favored response in patients with low ABCG2 score (Table 2).

**Figure 3:**
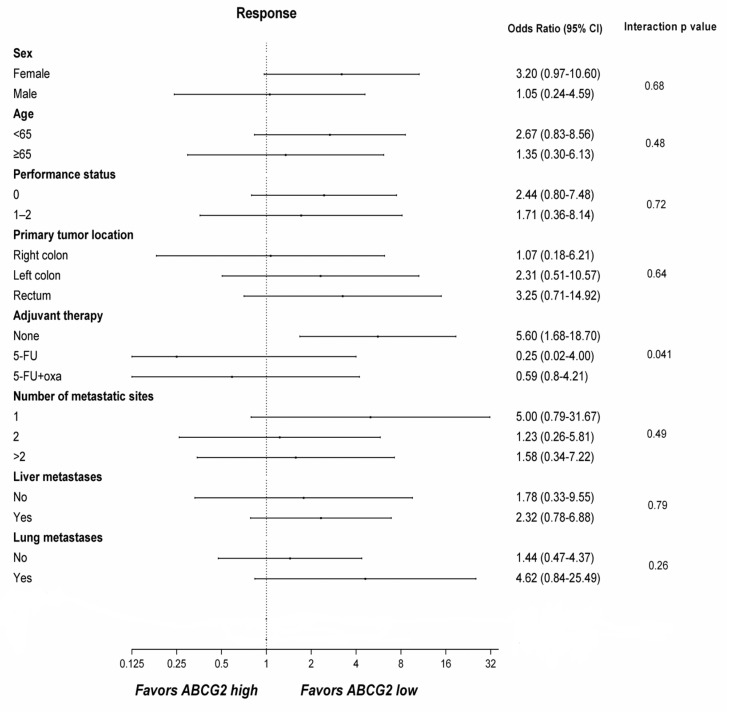
Exploratory predictive factor analyses of response to irinotecan containing treatment (*n* = 101).

In the exploratory interaction analysis, there was a significant interaction between the ABCG2 basolateral membrane score, prior adjuvant treatment, and OR (*p* = 0.041) as shown in Table 2 and Figure 3. The odds for response were greater in patients with low ABCG2 score and no prior adjuvant therapy (odds ratio: 5.6 (CI 1.68–18.7; *p* = 0.005)).

The ABCG2 basolateral membrane score was not associated with PFS or OS as the HRs for ABCG2 low vs. high were 1.21 (CI 0.81–1.82; *p* = 0.36) for PFS and 1.10 (CI 0.73–1.65; *p* = 0.65) for OS (Table 3). In the exploratory analyses for PFS and OS, ABCG2 low did not predict a significantly better outcome in patients with no prior adjuvant therapy (supplementary Figures S1 and S2). It is seen that patients who had received adjuvant oxaliplatin containing treatment had a significantly worse OS.

When the apical/luminal score was assessed two tumors (2%) were scored as 0, 11 (10%) tumors as 1+, 50 (46%) tumors as 2+, and 44 (41%) tumors as 3+, one tumor was not assessable. The ABCG2 apical/luminal membrane score was neither associated with OR, nor PFS nor OS as the odds ratio for OR for ABCG2 low vs. high was 0.79, *p* = 0.575; CI (0.75–2.72) and the HRs for PFS and OS for ABCG2 low vs. high were HR = 0.97, *p* = 0.89; CI (0.66–1.44) for PFS and 0.82, *p* = 0.35; CI (0.55–1.23) for OS. Likewise, analyses of the subgroup of patients with no prior adjuvant therapy revealed no significant associations between ABCG2 apical membrane score and OR, PFS, and OS.

Similar non-significant results were obtained when using the cytoplasmic ABCG2 score (data not shown). Figure S1 shows hazard ratio for progression-free survival in relation to ABCG2 (high/low expression), (n = 108). Figure S2 shows hazard ratio for overall survival in relation to ABCG2 level (n = 108).

## 3. Discussion

In this study we explored ABCG2 immunoreactivity as a candidate predictive biomarker for irinotecan used as first-line treatment for patients with mCRC. ABCG2 is a xenobiotic drug efflux pump, which is known to transport SN38 (the active metabolite of irinotecan) out of cancer cells thereby making the cancer cells less sensitive to irinotecan treatment [16]. We assessed the ABCG2 protein expression in order to test for any associations between biomarker status and treatment effects based on OR and survival data. Overall, our results did not support our main hypothesis that patients with tumors showing low ABCG2 protein expression would benefit the most from irinotecan containing therapy.

However, we did observe a trend for an association between ABCG2 immunoreactivity in the basolateral membrane of the cancer cells and OR in the logistic regression analysis, where the odds for response were greater for patients with low ABCG2 expressing tumors as compared to those with high ABCG2 expression. Furthermore, in exploratory analyses patients with ABCG2 low expressing tumors and no prior adjuvant treatment, the odds for response to irinotecan were 5.6 times greater (*p* = 0.005) also supported by a significant interaction between the ABCG2 basolateral membrane score, prior adjuvant treatment and OR (*p* = 0.041). In contrast, there was no association between ABCG2 expression and OR when the score for apical membrane or cytoplasmic immunoreactivity was included.

To our knowledge only two previous studies have investigated ABCG2 protein expression and outcome in patients with mCRC receiving irinotecan. The first study investigated the ABCG2 protein expression determined by IHC in 58 mCRC patients receiving 5-FU and irinotecan in the first-line setting. In contrast to our results, these authors were not able to demonstrate any association between ABCG2 expression and therapeutic outcome [20]. However, there are substantial methodological differences between the studies. Although the antibody used (BXP-21) was the same, detailed data regarding adjuvant treatment were not reported. Furthermore, the scoring system applied included cytoplasmic staining and membrane staining but did not differentiate between the basolateral and apical membranes. Thus, the results are not directly comparable with our study where we used a validated staining protocol and a pre-specified scoring protocol [19]. In a recent study by Trumpi et al. [21] protein expression of ABCG2 was analyzed by IHC in 566 patients with mCRC. The patients received irinotecan, either as second line monotherapy or as first line therapy in combination with capecitabine. Again, the authors found no significant association between ABCG2 cancer cell immunoreactivity and irinotecan response. However, only the luminal (apical) and cytoplasmic ABCG2 expression were assessed in this study. Thus, these two prior studies are in agreement with our results when including apical and cytoplasmic ABCG2 immunoreactivity only. However, 189 patients who underwent colorectal resection were enrolled in a retrospective study of ABCG2 protein expression determined by IHC (ABCG2 antibody: a mouse monoclonal antibody, BXP-21, Abcam Company, Cambridge, MA, USA). Seventeen patients received irinotecan-based chemotherapy for recurrent disease. In a multivariate logistic regression analysis, increased expression of ABCG2 was an independent predictor of resistance to SN-38. Furthermore, patients with increased levels of ABCG2 had shorter PFS than patients with low levels (104 versus 242 days; *p* = 0.047) [22].

In our study, we found no significant association between low and high ABCG2 immunoreactivity and PFS and OS. The exploratory analyses also failed to show that patients with low ABCG2 and no prior adjuvant treatment did have improved PFS or OS as one might have expected due to higher objective response rate in the ABCG2 low group of patients. One possible explanation for these results is that our data are purely coincidental but other possible explanations should also be considered. For example, the present study population represents a highly selected population due to the fact that they all were in conditions to allow for third-line therapy (this was a selection criterion for the total cohort). They thus constitute a group with an a priori favorable prognosis and good performance status, which perhaps to some extent could explain why OS was not significantly different between the ABCG2 groups.

We know that therapy can improve OR without influencing PFS and OS. This was for example reported in a subgroup analysis performed in the landmark trial CALGB/SWOG 80,405 including mCRC patients randomized between irinotecan/5-FU (FOLFIRI) and oxaliplatin/5-FU (FOLFOX6) with bevacizumab or cetuximab. For RAS wild type patients, the results showed a superior response rate in one arm but this was not translated into improved PFS or OS [23].

In CRC only two prior studies have reported high membranous expression of ABCG2 to be linked to poor prognosis. In one of these studies [24], high ABCG2 immunoreactivity was reported to correlate with the presence of lymph node metastases and in the other study [25] high ABCG2 immunoreactivity was associated with shorter OS. It is important to note that in the latter study, which investigated both the membrane (apical) and the cytoplasmic expression of ABCG2 protein in 225 primary CRC tissues, the authors only reported survival analysis for 69 patients. More recently, we have published on the association between *ABCG2* mRNA expression and irinotecan effects in the PETACC-3 prospective randomized clinical trial [13], where patients with stage III colon cancer were randomized to adjuvant 5FU/LV or 5FU/LV and irinotecan [8]. In that study a trend for a predictive effect of *ABCG2* mRNA expression as divided by the median *ABCG2* mRNA expression, was observed in relation to benefit of irinotecan containing therapy. However, when combined with another putative irinotecan predictive biomarker, *TOP-1* mRNA (*TOP-1* is the target for irinotecan) expression [26], a significant association with recurrence-free survival and OS were observed [13].

Another aspect is that the patient material in the present study is heterogeneous due to the fact that 40% had received prior adjuvant therapy. Prior adjuvant therapy may have influenced the results as studies have reported that ABCG2 expression may change after exposure to 5-FU and platinum containing therapy [27,28]. In the exploratory analyses for PFS and OS, ABCG2 low did not predict a significantly better outcome as patients who had received adjuvant oxaliplatin containing treatment had a significantly worse OS. One explanation to this result is that these patients did not have the opportunity to receive second-line treatment following the first-line irinotecan containing treatment and OS was calculated from the initiation of first-line treatment.

Moreover, whether changes in ABCG2 expression occur between primary tumor and metastases is not fully known. Only one study has reported on this subject by examining the mRNA expression in 10 primary CRC tumors and 39 corresponding hepatic metastases [11]. The authors suggest that the expression of ABCG2 can be altered between the primary and the metastatic lesions, but due to the study design no other conclusions could be drawn.

That prior treatment may alter expression of ABCG2 in cancer cells is supported by our recently published data on CRC cell lines made resistant to SN-38 by steadily increasing the dose of SN-38 [8]. It was seen that SN-38 resistance could be accompanied by a significant upregulation of the *ABCG2* mRNA and protein levels in the cells and a subsequent inhibition of the ABCG2 protein reversed the resistant phenotype.

Our study is limited by low statistical power. The relatively low number of included patients was reduced even further in the subgroup exploratory analyses. This calls for additional independent validation studies. However, our results from the adjuvant PETACC-3 study [13,29], strongly suggest a predictive value of ABCG2 mRNA expression in relation to adjuvant irinotecan treatment in chemo-naïve patients.

The strength of our study is the use of validated analytical methods where for ABCG2 IHC, the emphasis was on sensitivity, specificity and reproducibility of the ABCG2 antibody and a scoring protocol for the IHC data was pre-defined [19]. Furthermore, we included patients from a well-defined cohort of mCRC patients.

Many tyrosine kinase inhibitors (TKIs) are competitive or high affinity substrates of ABCG2, some of them such as sorafenib, sunitinib, lapatinib, erlotinib, gefitinib, imatinib, and nilotinib have been reported to be ABCG2 inhibitors or modulators. These TKIs seem to inhibit the function of ABCG2 by directly interacting with the substrate-binding sites, thus acting as competitive antagonists. In Scandion Oncology (www.scandiononcology.com) we have an ABCG2 inhibitor under clinical development. The drug, SCO-101, binds to ABCG2 and leads to its degradation. It also inhibits the function of the SRPK-1 kinase which has been suggested to be involved in drug resistance [30]. SCO-101 was in four clinical phase I studies shown to be a safe oral drug with only limited toxicity [31]. We have now initiated a clinical phase II study enrolling mCRC patients with acquired irinotecan resistance (the patients must have had a prior objective response to irinotecan following which they have progressed). The first part of the study is a dose-finding part where the recommended dose of SCO-1 when combined with FOLFIRI is established. The second part of the study will follow Simons Two Stage design [32]. Primary end-points are safety and efficacy (OR). For all patients, a fresh tumor biopsy will be obtained prior to treatment and this biopsy will be used for ABCG2 and SRPK-1 staining. In a post-treatment analysis, the immunohistochemical data will be associated with patient outcome.

## 4. Materials and Methods

### 4.1. Patients and Tumor Material

Patients were selected retrospectively from a Danish national cohort of 498 patients with mCRC. All patients in this cohort had received irinotecan in combination with cetuximab in third line at the Departments of Oncology, Herlev, Odense, and Aalborg Hospitals, Denmark. We selected from this cohort those patients who had received first-line treatment with irinotecan containing drugs. The inclusion period was specified as 1 January 2005 to 1 August 2008; this time interval was selected specifically because it preceded the introduction of KRAS genotyping. Accordingly, mutational status of KRAS, NRAS, and BRAF was unknown. The present study cohort includes 119 patients, who all had received one of the following regimens as first-line therapy, irinotecan, 5-FU/LV + irinotecan, capecitabine + irinotecan, or 5-FU/LV + irinotecan + bevacizumab.

Objective response (OR) data and survival data were obtained from all patients by retrospective chart review. According to RECIST 1.0 [33], OR data was based on the computed tomography (CT) scans of the thorax and abdomen that were performed every 9–12 weeks during the treatment period [33]. The present study was approved by the Research Ethics Committee of Copenhagen (H-KA-20060094). Reporting of the results was prepared according to the REMARK guidelines [34].

Formalin-fixed, paraffin-embedded (FFPE) primary tumor material was collected retrospectively. The samples originated from the primary surgical resection specimens or endoscopic biopsies. H&E stained sections were reviewed by JAP and DL for diagnostic confirmation of adenocarcinoma.

### 4.2. ABCG2 Immunohistochemistry

ABCG2 IHC was conducted on whole sections cut at 3 µm and mounted on SuperFrostTM Plus slides just prior to analysis. We recently thoroughly validated six anti-ABCG2 antibodies for IHC and the conclusion was that the ABCG2 antibody BXP-21 (Abcam, Cambridge, UK) performed superior to the other tested antibodies [19]. In brief, sections were deparaffinized in xylene and rehydrated in a series of graded ethanol. Heat induced antigen retrieval (HIER) was performed with Target Retrieval solution, pH 9 (Dako, Glostrup, Denmark). Sections were allowed to cool in solution for 20 min at room temperature (RT) before endogenous peroxidase was blocked in a 1% hydrogen peroxide solution for 10 min prior to incubation with the ABCG2 antibody BXP-21 for 1 h at RT. BXP-21 was diluted 1:500 in ready-to-use antibody diluent with background reducing agents (Dako, Glostrup, Denmark). The antigen-antibody reactions were visualized with the chromogen 3,3’-diaminobenzidine-tetrahydrochloride (DAB+, Dako, Glostrup, Denmark) followed by counterstaining with Mayer’s Hematoxylin. Assessment of immunoreactivity was primarily based on basolateral membrane staining and followed the guidelines described for HER2 protein testing in gastric cancer [35]. The staining was classified as 0 (no expression in > 90% cancer cells, at 40× magnification), 1+ (weak expression in ≥ 10% cancer cells, only visible at 40× magnification), 2+ (weak to moderate expression in ≥ 10% cancer cells, visible at 10/20× magnification), 3+ (strong expression in ≥10% cancer cells, visible at 4× magnification). Furthermore, the apical membrane staining and cytoplasmic immunostaining were given separate scores using the same guidelines. Assessment of immunostaining was conducted separately by SBN and BMV. In cases of ambiguity the final score was achieved by consensus.

### 4.3. Statistics

The primary endpoint was OR. OR was dichotomized in response and no-response, where response was defined as complete response (CR) or partial response (PR) and no-response as stable disease (SD) or progressive disease (PD). Secondary endpoints were progression-free survival (PFS) and overall survival (OS). PFS was defined as time from start of treatment to disease progression or death from any cause. OS was defined as time from start of treatment to death from any cause. The latest follow-up on survival data was performed in October 2014 and events after this date were censored. Fisher’s exact test was used to test for associations between grouped baseline characteristics and ABCG2 protein expression. The scores for the immunoreactivity of ABCG2 expression were dichotomized and labeled “low” (0, 1+ or 2+) or “high” (3+). The Cox proportional hazards (CPH) regression model was used for survival analysis. Results were presented by hazard ratios (HR) with 95% confidence interval (CI). Model assessment was evaluated using graphical methods, log (-log (survival)). Multivariate analysis was performed only if the results from univariate analysis were significant.

The association between OR and biomarker status was analyzed using logistic regression. Patients with non-evaluable disease according to RECIST 1.0 were excluded. Odds ratios and HR with 95% CI were reported. The Hosmer–Lemeshow test was applied for assessment of goodness of fit. The predefined hypothesis was that mCRC patients having low apical membrane ABCG2 immunoreactivity in their cancer cells benefit the most from irinotecan containing therapy.

We also performed explorative analyses to identify predictive factors using logistic regression and CPH models entering the dichotomized value of ABCG2 (biomarker), a potential predictive factor (baseline characteristics), and a biomarker-predictive factor interaction term. Correction for multiple testing was not done in the explorative analyses.

Results with *p*-values < 0.05 were considered statistically significant. All calculations were performed using IBM SPSS statistics 22.0.

## 5. Conclusions

We found a significant association between ABCG2 basolateral immunoreactivity in the cancer cells and response to irinotecan containing therapy in chemo-naïve patients with mCRC. Validated predictive biomarkers for irinotecan may in the future become valuable tools for clinicians when deciding first-line treatment for patients with mCRC. ABCG2 status, alone or in combination with other biomarkers, e.g., *TOP-1*, may have the potential of use in daily clinical practice. However, before any firm conclusion can be drawn our results must be reproduced in larger and independent studies including well-defined cohorts of patients with mCRC. 

## Figures and Tables

**Table 1:** Patient characteristics.

		*Total* *N* (%)	ABCG2 Low N (%)	ABCG2 High N (%)	Fisher’s Exact Test *p*
Patients included		*108 (100)*	72 (67)	36 (33)	
Gender	Males	61 (56)	37 (61)	24 (39)	0.15
	Females	47 (44)	35 (74)	12 (26)	
Age	<65	70 (65)	44 (63)	26 (37)	0.29
	≥65	38 (35)	28 (74)	10 (26)	
WHO PS	0	71 (66)	46 (65)	25 (35)	0.67
	1–2	37 (34)	26 (70)	11 (30)	
Location primary tumor	Right	30 (28)	19 (63)	11 (37)	0.84
	Left	40 (37)	28 (70)	12 (30)	
	Rectum	38 (35)	25 (66)	13 (34)	
Primary tumor resected	Yes	98 (91)	62 (63)	36 (37)	0.029
	No	10 (9)	10 (100)	0 (0)	
Prior radiotherapy	Yes	9 (8)	5 (56)	4 (44)	0.48
	No	99 (92)	67 (68)	32 (32)	
*Adjuvant chemotherapy*	No	65 (60)	42 (65)	23 (35)	0.43
	5-FU	14 (13)	8 (57)	6 (43)	
	5-FU + Oxa	29 (27)	22 (76)	7 (24)	
Number of metastatic sites	1	33 (30)	24 (73)	9 (27)	0.36
	2	42 (39)	29 (69)	13 (31)	
	>2	30 (28)	17 (57)	13 (43)	
	*Unknown*	3 (3)			
Liver metastases	Yes	31 (29)	18 (58)	13 (42)	0.26
	No	77 (71)	54 (70)	23 (30)	
Lung metastases	Yes	39 (36)	25 (64)	14 (36)	0.68
	No	69 (64)	47 (68)	22 (32)	

Abbreviations: 5-FU, 5fluorouracil; Oxa, oxaliplatin; Bev, bevacizumab; HR, hazard ratio; CI, confidence interval.

**Table 2:** Objective response according to biomarker (high/low expression).

			Response	No-Response	Response vs. No-Response		
		N (%)	N (%)	N (%)	OR	95% CI	*p*
Study population ^a^		101 (100)	43 (43)	58 (57)			
ABCG2	Low	71 (70)	34 (48)	37 (52)	2.14	0.86–5.32	0.10
	High	30 (30)	9 (30)	21 (70)	(ref) ^b^		

^a^ Assessable for response; ^b^ Used as reference value.

**Table 3:** Univariate survival analyses of patient characteristics.

		*Total N* (%)	Progression-Free Survival				Overall Survival			
	
		*108 (100)*	HR		95% CI	*p*	HR		95% CI	*p*
Gender	Males	61 (56)	1.08	0.73–1.60		0.70	0.92	0.62–1.35		0.66
	Females	47 (44)	(ref)				(ref)			
Age	Per 10 year increase		1.06	0.86–1.30		0.59	1.04	0.84–1.28		0.75
WHO PS	0	71 (66)	0.69	0.45–1.04		0.07	0.74	0.49–1.10		0.14
	1–2	37 (34)	(ref)				(ref)			
Location primary tumor	Right	30 (28)	0.78	0.48–1.29		0.34	0.97	0.60–1.60		0.91
	Left	40 (37)	1.18	0.75–1.85		0.47	1.19	0.76–1.87		0.46
	Rectum	38 (35)	(ref)				(ref)			
Primary tumor resected	Yes	98 (91)	0.8	0.41–1.53		0.49	0.79	0.41–1.52		0.48
	No	10 (9)	(ref)				(ref)			
Prior radiotherapy	Yes	9 (8)	(ref)				(ref)			
	No	99 (92)	0.52	0.26–1.04		0.07	0.6	0.30–1.21		0.15
Adjuvant chemotherapy	No	65 (60)	(ref)				(ref)			
	5-FU	14 (13)	1.5	0.82–2.75		0.19	1.2	0.67–2.15		0.54
	5-FU + Oxa	29 (27)	2.62	1.63–4.22		<0.001	2.16	1.37–3.39		0.001
Number of metastatic sites	1	33 (30)	(ref)				(ref)			
	2	42 (39)	1.07	0.67–1.70		0.78	1.3	0.81–2.07		0.27
	>2	30 (28)	1.33	0.80–2.21		0.27	2.00	1.18–3.36		0.010
	*Unknown*	3 (3)								
Liver metastases	Yes	31 (29)	1.00	0.66–1.53		0.99	1.23	0.80–1.87		0.34
	No	77 (71)	(ref)				(ref)			
Lung metastases	Yes	39 (36)	0.96	0.64–1.44		0.85	1.08	0.73–1.62		0.7
	No	69 (64)	(ref)				(ref)			

Unknowns were not included in the shown proportions or test for distribution differences. Abbreviations: ref, used as reference value; 5-FU, 5-fluorouracil; Oxa, oxaliplatin; Bev, bevacizumab; HR, hazard ratio; CI, confidence interval.

## Supplementary Materials

Supplementary Materials can be found at https://www.mdpi.com/1422-0067/21/14/5027/s1. Figure S1: Analyses showing hazard ratio for progression-free survival in relation to ABCG2 (high/low expression), (*n* = 108). Figure S2: Analyses showing hazard ratio for overall survival in relation to ABCG2 level (*n* = 108).

## Abbreviations

5-FU	5-fluorouracil
Oxa	oxaliplatin
Bev	bevacizumab
HR	hazard ratio
CI	confidence interval
CRC mCRC TOP1	Colorectal cancer Metastatic Colorectal Cancer Topoisomerase 1 enzyme
ABC	ATP-binding cassette
