# Supplementary material for: ABCG2 Protein Levels and Association to Response to First-Line Irinotecan-Based Therapy for Patients with Metastatic Colorectal Cancer
Source: Int J Mol Sci. 2020 Jul 16;21(14):5027. doi: 10.3390/ijms21145027 (PMC7404184; doi:10.3390/ijms21145027)
Supplement: Supplementary file 1 [file ijms-21-05027-s001.pdf]

Supplementary

Figure S1

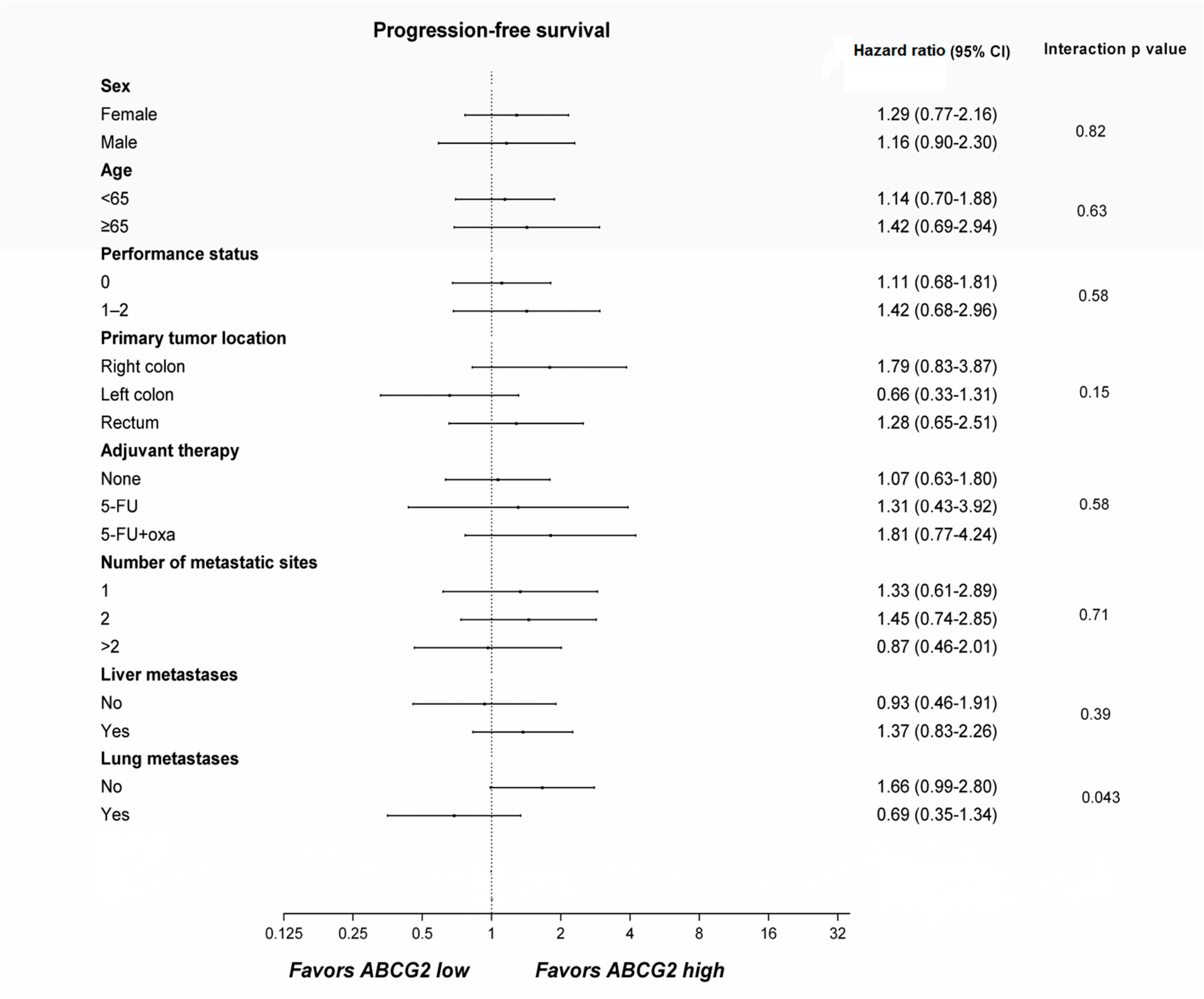

Figure S1

Analyses showing hazard ratio for progression-free survival in relation to ABCG2 (high/low expression), (n= 108). Interaction p values: test for interaction between the biomarker and clinical variables.

Figure S2

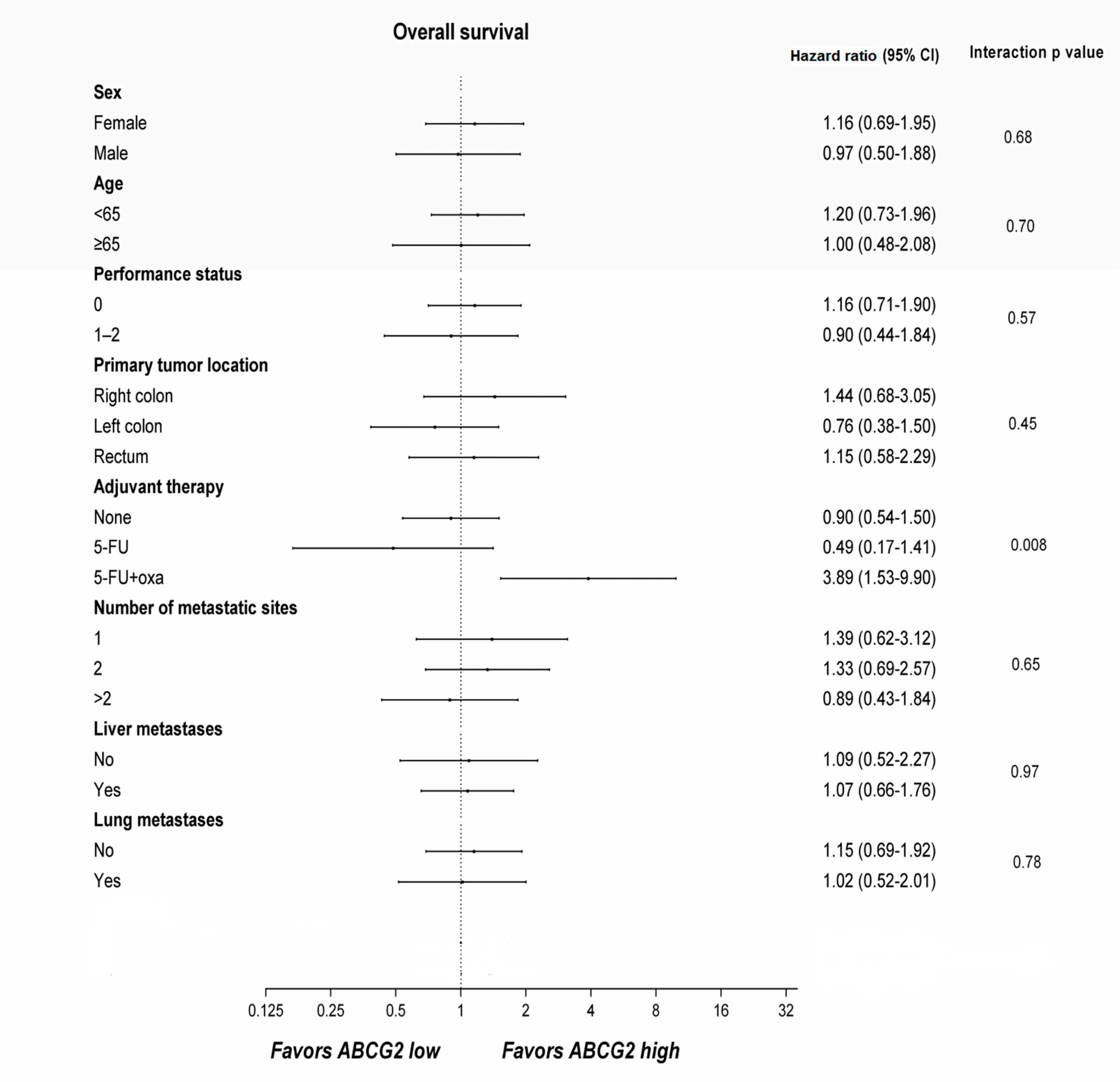

Figure S2

Analyses showing hazard ratio for overall survival in relation to ABCG2 level (n= 108). Interaction p values: test for interaction between the biomarker and clinical variables.
